# Multi-Joint Analysis of Pose Viability Supports the Possibility of Salamander-Like Hindlimb Configurations in the Permian Tetrapod *Eryops megacephalus*

**DOI:** 10.1093/icb/icac083

**Published:** 2022-06-10

**Authors:** Eva C Herbst, Armita R Manafzadeh, John R Hutchinson

**Affiliations:** Palaeontological Institute and Museum, University of Zurich, Karl-Schmid-Strasse 4, CH-8006 Zurich, Switzerland; Structure and Motion Lab, Department of Comparative Biomedical Sciences, The Royal Veterinary College, Hawkshead Lane, Hertfordshire AL9 7TA, UK; Department of Ecology, Evolution, and Organismal Biology, Brown University, 80 Waterman Street, Providence, RI 02912, USA; Structure and Motion Lab, Department of Comparative Biomedical Sciences, The Royal Veterinary College, Hawkshead Lane, Hertfordshire AL9 7TA, UK

## Abstract

Salamanders are often used as analogs for early tetrapods in paleontological reconstructions of locomotion. However, concerns have been raised about whether this comparison is justifiable, necessitating comparisons of a broader range of early tetrapods with salamanders. Here, we test whether the osteological morphology of the hindlimb in the early tetrapod (temnospondyl amphibian) *Eryops megacephalus* could have facilitated the sequence of limb configurations used by salamanders during terrestrial locomotion. To do so, we present a new method that enables the examination of full limb configurations rather than isolated joint poses. Based on this analysis, we conclude that *E. megacephalus* may indeed have been capable of salamander-like hindlimb kinematics. Our method facilitates the holistic visual comparison of limb configurations between taxa without reliance on the homology of coordinate system definitions, and can thus be applied to facilitate various comparisons between extinct and extant taxa, spanning the diversity of locomotion both past and present.

## Introduction

The origin of terrestrial locomotion is one of the key transitions in vertebrate history, but we still do not know how most early tetrapods moved on land. To address this question, paleontologists have often turned to salamanders as an analog due to similarities in morphology and presumed habitat (Schaeffer 1941; Ashley-Ross 1994; Kawano and Blob 2013). However, several studies have suggested that salamanders may not always be the most appropriate model for early tetrapod locomotion. For example, analyses of osteological joint mobility have concluded that the stem tetrapod *Ichthyostega*, unlike a salamander, was unable to draw its pes beneath its body to contact the substrate with the plantar surface of the foot (Pierce et al. 2012). Similarly, an integration of joint range of motion (RoM), dynamic simulations, a robot model, trackway information, and comparisons with extant taxa indicated that the stem amniote *Orobates* may have walked more like a caiman (i.e., more erect) than a salamander (Nyakatura et al. 2019). As a result, further biomechanical studies spanning a broader range of early tetrapods are needed to test whether some of these animals may have been capable of a salamander-like gait, or whether this comparison is truly uninformative across the board.

**Fig. 1:**
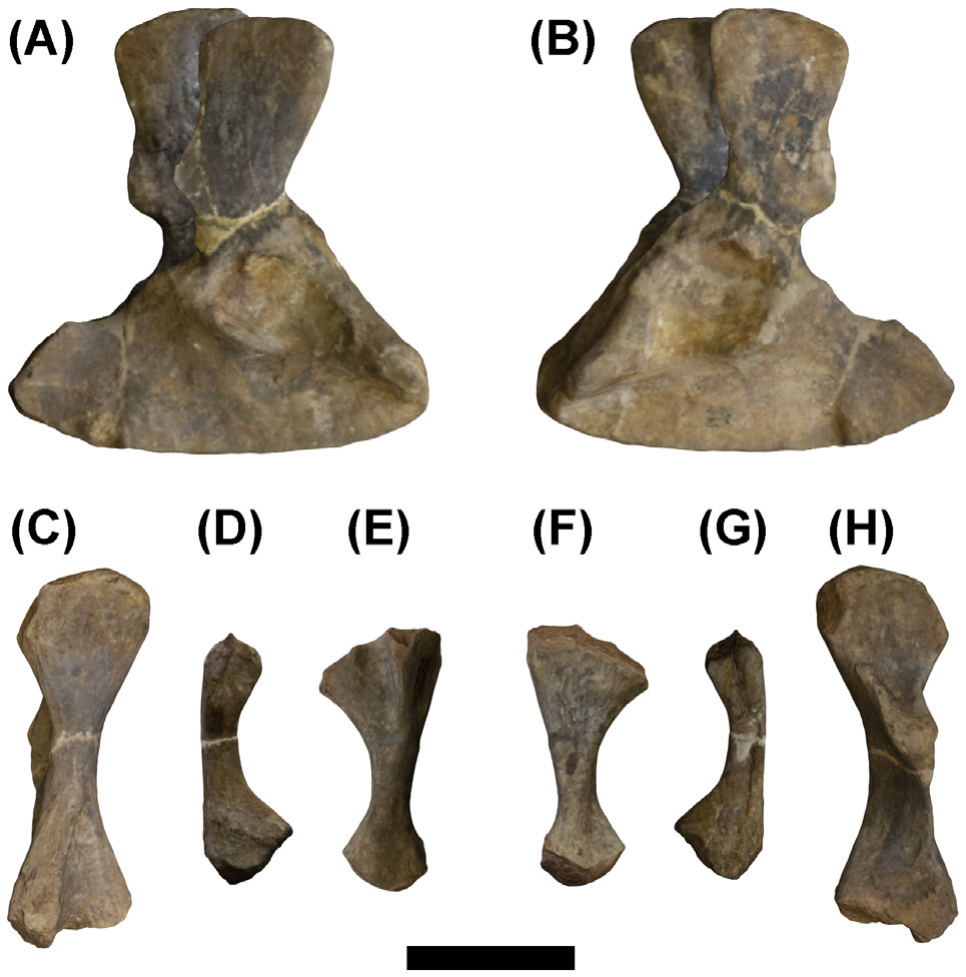
*Eryops megacephalus* photogrammetry models, scaled to fit limb proportions of a single individual. Pelvis FMNH UC 446 in **(A)** right lateral and **(B)** left lateral views. **(C)** Right femur FMNH UC 33 in a lateral view; **(D)** right fibula FMNH UC 203 in a lateral view; **(E)** right tibia FMNH UC 1250 in a lateral view; **(F)** right tibia FMNH UC 1250 in a medial view; **(G)** right fibula FMNH UC 203 in a medial view; and **(H)** right femur FMNH UC 33 in a medial view. Scale bar = 10 cm.

“Early tetrapods” includes stem tetrapods such as *Acanthostega* and *Ichthyostega*, which seem to have retained some of their ancestral aquatic specializations, as well as crown-group Tetrapoda, including stem temnospondyls (Clack 2002b). Temnospondyli have been proposed to include crown group Lissamphibia (extant amphibians), forming the sister group to Amniotes (Sigurdsen and Green 2011; Pardo et al. 2017, but see Marjanovic and Laurin 2019 for data supporting the lepospondyl origin of Lissamphibia). One early tetrapod displaying seemingly strong similarities with salamanders is the Permian tetrapod *Eryops megacephalus* Cope 1877, a large temnospondlyl known from many fossils. Shared morphological features between *E. megacephalus* and salamanders include a shallow acetabulum (Fig. 1) and an enlarged capitulum on the humerus, the latter of which is characteristic of lissamphibians and some temnospondyls (Sigurdsen and Bolt 2009). Furthermore, these animals have very similar limb segment proportions, sharing a tibia/femur length ratio of about 0.6. Granted, salamanders and *E. megacephalus* are quite different in some ways that could alter locomotion, especially the very large size of the latter (~3 m adult body length; Schoch 2002). Testing whether *E. megacephalus* is capable of walking like a salamander would therefore illuminate whether living salamanders may still be an appropriate model for some early tetrapods. If *E. megacephalus* appears incapable of a salamander-like walk, then early tetrapods less morphologically similar to salamanders would likely also have been incapable. However, if *E. megacephalus* appears capable of salamander-like limb configurations, then salamanders may still be an informative analog for the locomotion of some early tetrapods.

Here, we test whether the hindlimb of *E. megacephalus* may have been capable of salamander-like hindlimb configurations using data from (1) osteological simulations of joint mobility in *E. megacephalus* (following Manafzadeh and Padian 2018; Manafzadeh and Gatesy 2021) and (2) *in vivo* Fire salamander (*Salamandra salamandra*) walking data (Herbst et al. in review). To account for the interacting effects of joint mobility and limb segment proportions (Gatesy and Pollard 2011), we present a novel method using a “digital marionette” to evaluate full-limb configuration viability by checking the osteological viability of multiple joints’ poses at once. We then use this approach to test whether key limb configurations in the stride cycle of *S. salamandra*can be assumed by the hindlimb of *E. megacephalus*. Ultimately, we discuss the implications of this work for future paleontological reconstructions of locomotion.

## Materials and methods

### Specimens and photogrammetry models

We examined all available *E. megacephalus* material in the collections at the Cambridge Museum of Zoology (UMZC) and the Field Museum in Chicago (FMNH). We chose specimens for photogrammetry (details in Supplementary Information) based on preservation (completeness and quality of element, especially the articular surfaces), to avoid taphonomic artifacts such as distortion or abrasion of articular surfaces. Between the two museums, no single individual with a complete and well-preserved hindlimb exists. Therefore, we selected specimens and scaled the models based on data from the literature (Cope 1880, 1888; Pawley and Warren 2006; details in Table 1, Supplementary Text 1 and Supplementary Table S1) so that the proportions of the pelvis, femur, tibia, and fibula were realistic. Specimens used were FMNH UC 446 (pelvis), FMNH UC 33 (femur), FMNH UC 203 (fibula), and FMNH UC 1250 (tibia) (Fig. 1). The pelvis was somewhat flattened but primarily on the left side (except some slight distortion in the dorsal iliac process, which would not affect osteological RoM analyses); therefore, we used the right side for our RoM analyses (see Supplementary Text 1 for more information).

**Table 1:** *Eryops megacephalus* hindlimb proportions from the literature, and *S. salamandra* measurements from our rotoscoping specimen (length is maximum length in the given dimension)

	Elements	Measurement dimensions	Ratio	Source
*E. megacephalus*	Femur : pelvis	Proximodistal length : anteroposterior length	0.92	Cope 1880, Plate IV
*E. megacephalus*	Tibia : femur	Proximodistal length : proximodistal length	0.62	Pawley and Warren 2006, Figs. 10 and 11
*E. megacephalus*	Fibula : tibia	Proximodistal length : proximodistal length	0.98	Cope 1888, Text
*S. salamandra*	Femur : pelvis	Proximodistal length : anteroposterior length	0.62*	Own measurements
*S. salamandra*	Tibia : femur	Proximodistal length : proximodistal length	0.57	Own measurements
*S. salamandra*	Fibula : tibia	Proximodistal length : proximodistal length	0.85	Own measurements

* Note: *S. salamandra* pelvis is not fully ossified anteriorly.

We then used photogrammetry to create 3D digital models of the bones. We took between 119 and 320 photos for each half of each specimen, ensuring that all angles of view of the object were sufficiently sampled in the photographs. Using the photogrammetry software AgiSoft Metashape (AgiSoft PhotoScan Standard, Version 1.5.4, 201, retrieved from http://www.agisoft.com/downloads/installer/), we aligned these photos (settings: high accuracy, generic preselection, adaptive camera model fitting, 50,000 key point limit, and 4000 tie point limit) and produced a dense point cloud (settings: high quality and aggressive filtering) and subsequently a mesh of both sides of each element. We then manually cleaned the model in AgiSoft, removing the background. Measurements and scaling were performed in MeshLab software (Meshlab v1.3.3, Cignoni et al. 2008). The final scaled models of the *E. megacephalus* hindlimb are displayed in Fig. 1. For information on salamander CT scans and rotoscoping data, see Herbst et al. (in review).

### Rigging and osteological RoM simulation

We first implemented the RoM reconstruction methods developed by Manafzadeh and Padian (2018) and Manafzadeh and Gatesy (2021) to test the osteological RoM allowed by the hip and knee joints of *E. megacephalus*. We created right-sided digital marionettes (i.e., forward kinematic rigs) for both joints in Maya (versions 2019 and 2022, Autodesk, San Rafael, CA, USA) and then animated each joint in 5 degree increments in all three rotational degrees of freedom (flexion–extension [FE], abduction–adduction [ABAD], and long-axis rotation [LAR]) to automatically sample all possible combinations of rotations (186,624 unique poses). At each frame of animation, we assessed pose viability by checking for a Boolean intersection (i.e., interpenetration of distal and proximal bone meshes); if there was no intersection, the pose was marked as viable (Manafzadeh and Padian 2018). Maya Embedded Language (MEL) scripts for such osteological RoM sampling can be found in Manafzadeh and Gatesy (2022).

**Fig. 2:**
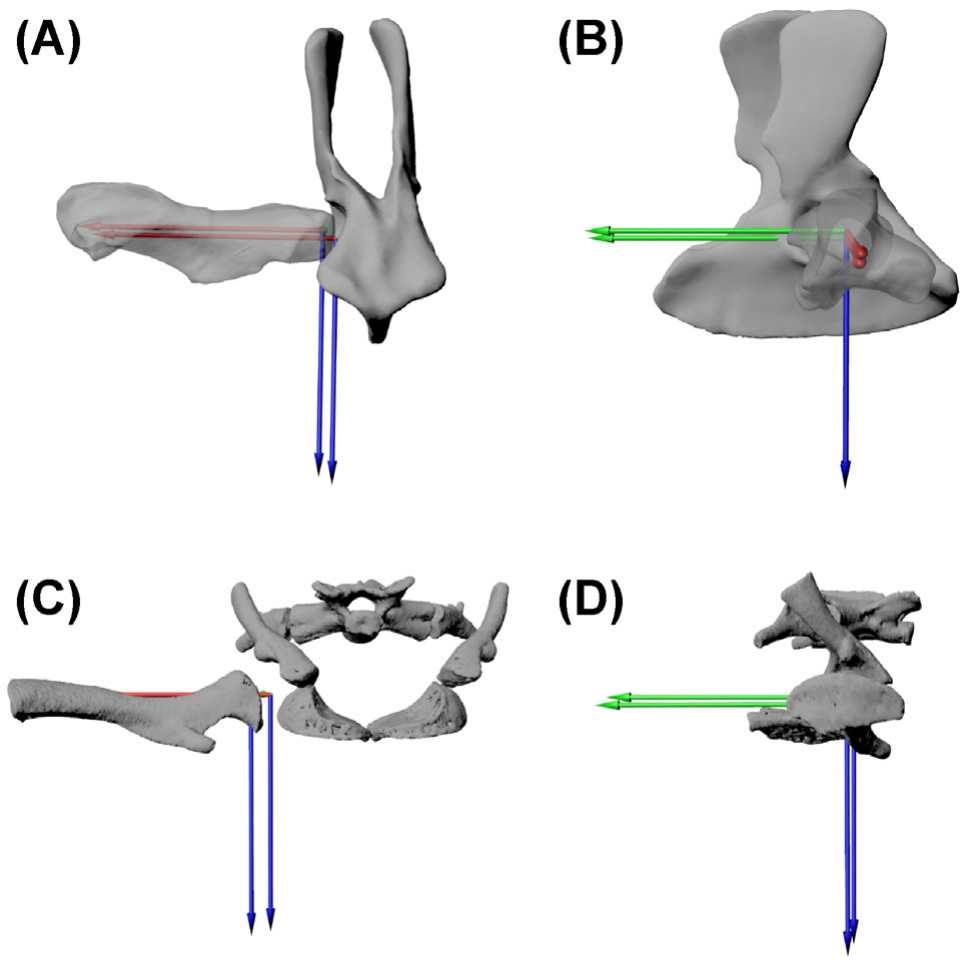
*Eryops megacephalus* right hip joint in an **(A)** anterior view and **(B)** lateral view, with right acetabular ACS and right proximal femur ACS in null pose (with femur laterally extended). The proximal femur ACS was translated slightly laterally and dorsally to account for cartilage and to prevent the femur intersecting with the ventral acetabular rim (translation values discussed in text). The animation joint in Maya was positioned mid-way between the two ACS origins with a point constraint, and the axes of the joint were aligned with the axes of the ACSs in null pose with an orient constraint. (**C** and **D**) *Salamandra salamandra* hip joint in anterior and lateral views. The *Z*-axis (FE, blue) points ventrally, the *Y*-axis (ABAD, green) points posteriorly, and the *X*-axis (LAR, red) points laterally. Not to scale.

To set up the hip joint, we aligned the proximal femoral anatomical coordinate system (ACS) to the right acetabular ACS (Fig. 2; see Supplementary Text 1 and Supplementary Fig. S1 for ACS creation information). The ACSs were developed using the same methodology as used for the salamander (Herbst et al. in review) to allow standardized comparisons between species. For the proximal femoral ACS of *E. megacephalus*, we subsequently rotated the ACS by 90 degrees about the LAR axis. This reorientation was necessary because our ACSs were defined based on proximal femoral morphology, and *S. salamandra* and *E. megacephalus* differ in proximal femur morphology relative to the distal end; this relationship is often termed femoral “torsion.” In *E. megacephalus*, the long dimensions of the distal femur and proximal femur are roughly aligned, whereas in *S. salamandra* they are 90-degrees offset (Fig. 2). The ACS rotation enabled us to ensure that in *E. megacephalus* and *S. salamandra* the distal femur and tibia/fibula orientations are similar between the taxa in the null pose and have the same spatial relationships to the femoral ACS in both taxa.

To account for cartilage on the surface of the bones, we then translated the femur 10.83 mm laterally (along the *X*-axis) and 6.28 mm dorsally (along the −*Z*-axis), centering the proximal femur within the acetabulum. We then placed a Maya animation joint in the center of the joint space (between the proximal and distal ACSs) and oriented it to the ACSs using animation constraints (Fig. 2).

To set up the knee joint, we first orient- and point-constrained the proximal tibia/fibula ACS to the ACS of the distal femur (following Kambic et al. 2014). We then created a double animation joint positioned and oriented to these ACSs (the parent joint for position and the child joint for joint rotations). We used the prism-based hinge joint protocol developed by Manafzadeh and Gatesy (2021) to create joint spacing that remains more constant throughout rotations of the joint, which would not have been possible with a simple rotational joint in this case. In the salamander joint, the simple rotational animation joint was used in combination with added translations as needed at some poses to track the bone movement; however, this would not have been feasible for *E. megacephalus*, as we needed to include such translations before the osteological RoM tests.

**Fig. 3:**
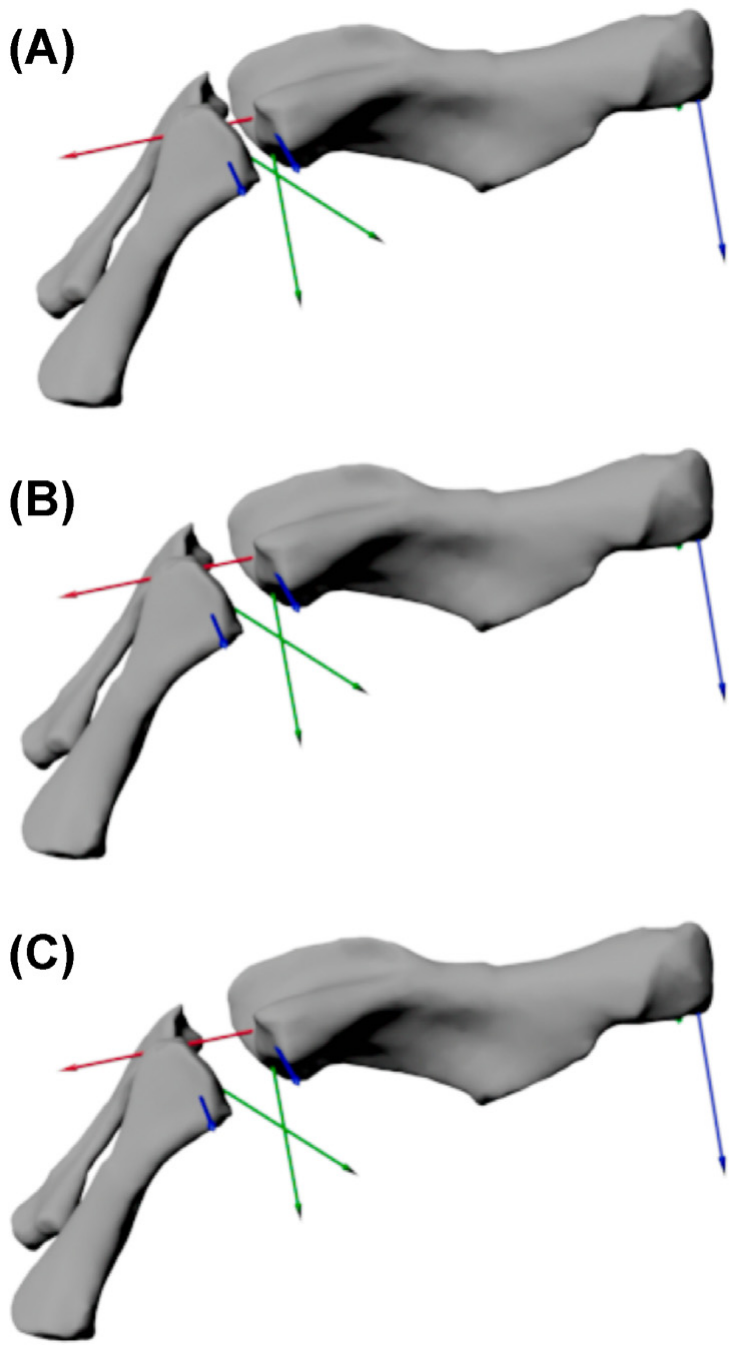
*Eryops megacephalus* right knee joint in anterior view, flexed (rotated about *Z*) at 45 degrees from the null pose (in which the proximal tibia/fibula ACS is aligned with the distal femoral ACS). **(A)** Knee spacing option A (tight joint spacing); **(B)** knee spacing option B (intermediate joint spacing); and **(C)** knee spacing option C (large joint spacing, based on the same relative joint spacing as in *S. salamandra*in the null pose).

The joint spacing was determined by positioning the joint in 45-degree FE (i.e., 45-degrees flexed from the null pose where the knee is extended; a position chosen to objectively assign joint configuration without biasing extremely flexed or extended poses) and adjusting translations until the articular surfaces were aligned. This is referred to as *E. megacephalus* knee B (Fig. 3 B) (letters refer to knee joint tightness in ascending order). To quantify the spacing, we measured the distance in local X from the distal femoral ACS to the proximal tibiofibular ACS (Supplementary Fig. S2). This distance was 2.9 cm. Note that since the ACSs are placed slightly below the bone surface, the actual joint “space” is slightly smaller.

As a sensitivity study to determine how different joint spaces affect the osteological joint RoM (i.e., inferring different cartilage thicknesses; Holliday et al. 2010; Molnar 2021), we created two other models in addition to our initial model.

*Eryops megacephalus* knee C joint spacing was a model in which the joint spacing was based on relative joint spacing in our salamander model, which is based on *in vivo* joint spacing (Fig. 3 C). We positioned the salamander model into a fully extended limb pose (zero rotations about all axes) and measured the distance between the distal femoral ACS and proximal tibia/fibula ACS along the *X*-axis. This distance was 26% of the tibial length (longest proximodistal direction). We then created this same joint spacing (relative to tibia length) between the ACSs of the *E. megacephalus* knee joint. To determine the translations along the other axes, as in knee B, we rotated the knee joint to 45 degrees and then adjusted the bones so that the articular surfaces lined up. To compare the joint spacing with model A, we used the same method as above, measuring the spacing at the null pose: the distance between ACSs along the *X-*axis was 3.59 cm. Again note that since the ACSs are placed slightly below the bone surface, the actual joint “space” is slightly smaller. *Eryops megacephalus* knee joint A was a model in which we added a tighter joint spacing (Fig. 3 A) than in models B and C, to test the effects of this on the RoM. The distance between the distal femoral ACS and proximal tibia/fibula ACS was 2 cm in this model.

**Fig. 4:**
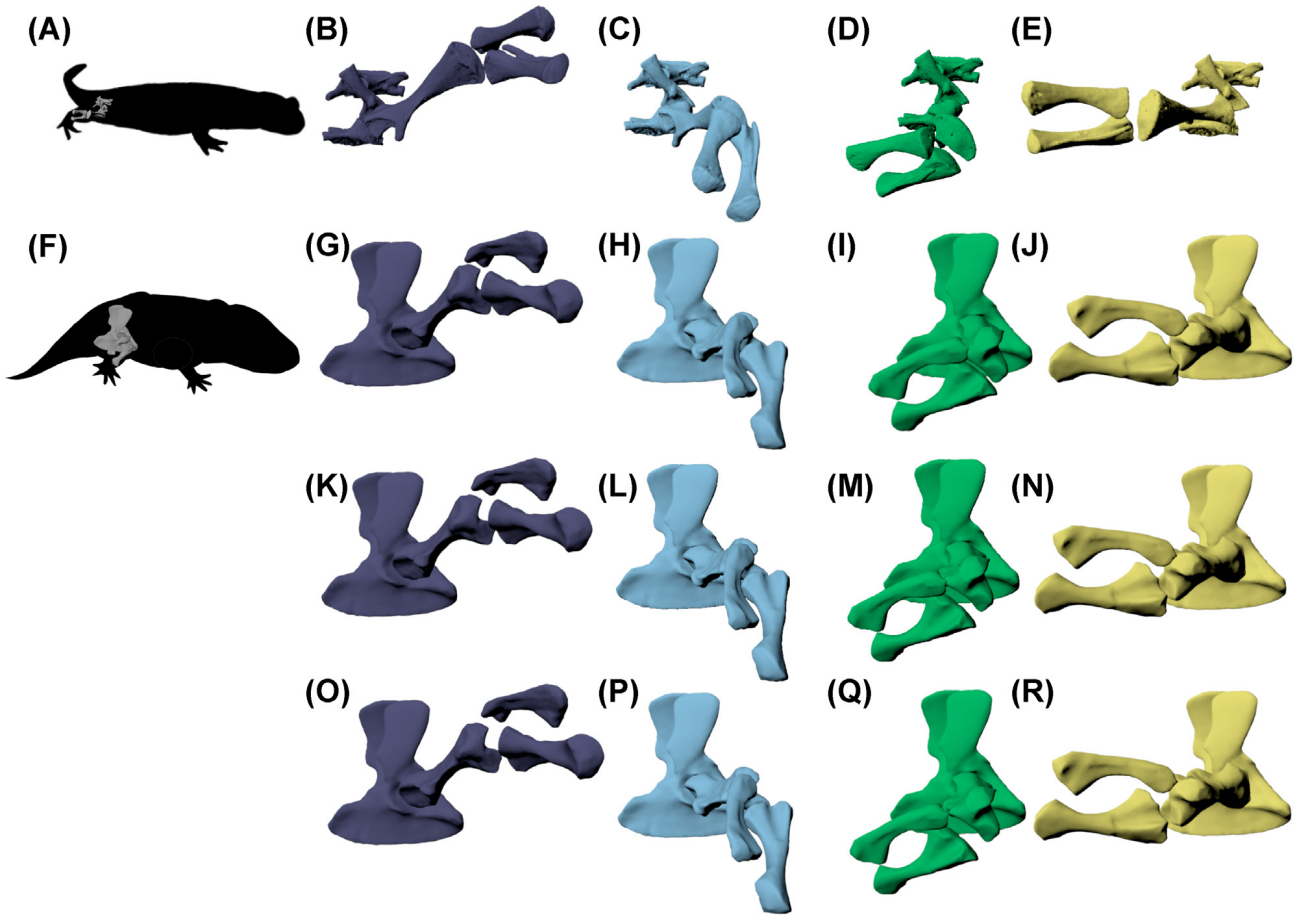
Hindlimb configurations in *S. salamandra***(A)** from rotoscoping of *in vivo* walking, during **(B)** mid-swing, **(C)** toe-on, **(D)** mid-stance, and **(E)** just before toe-off. These limb configurations were recreated in *E. megacephalus***(F)** with three different knee spacing options: **(G–J)** tight knee spacing; **(K–N)** intermediate knee spacing; and **(O–R)** larger knee spacing, based on the amount of knee spacing present in the rotoscoped salamander at the null pose. *Salamandra salamandra* configurations in (B–E) were scaled to *E. megacephalus* knee B.

We used scientific rotoscoping (a method in which CT scanned bones of an animal are aligned to two biplanar X-ray videos to obtain 3D bone motion [Gatesy et al. 2010]) to determine the hip and knee joint poses and full hind limb configurations used during a walking stride in the Fire salamander (*S. salamandra*) (Herbst et al. in review). The relative hindlimb bone proportions are given in Table 1. We chose four key hind limb configurations to re-create in *E. megacephalus*: mid-swing (i.e., maximal anterior excursion of the limb, frame 565), toe-on (frame 636), mid-stance (frame 716), and toward the end of the stance, just before toe-off (frame 852) (500 Hz recording) (Fig. 4 B–E). We chose these poses to represent various points of the stride cycle, to show a range of limb orientations in the salamander hindlimb during walking. Furthermore, we picked these poses in the salamander independently of the viable pose space in *E. megacephalus*, to prevent biasing pose choice toward poses we observed in *E. megacephalus*.

### A new multi-joint method for testing limb configuration viability

We devised a new method combining osteological RoM (graphed as Euler alpha shapes [polygonal envelopes representing the bounds of the point cloud of viable poses]; Manafzadeh and Padian 2018) with a forward kinematic model whose joints could be interactively manipulated. This enabled us to recreate key hindlimb configurations used in *S. salamandra* during walking (Herbst et al. in review) in the early tetrapod *E. megacephalus*. The method then gives automatic feedback whether the hip and knee poses required for this hindlimb configuration are viable, based on the osteological RoM data. This setup also enabled us to test the sensitivity of our results to the knee joint spacing. The advantage of this method over simply comparing possible joint angles was that we took into consideration the whole limb morphology and the contributions of both the hip and knee to various limb configurations, and obtained results independent of the specifics of our joint coordinate system definitions. This is important because the morphology of *S. salamandra* and *E. megacephalus* is not exactly the same (although they do share the same femur to tibia length ratio).

We created a rig in Maya that included both the hip and knee joints and the pelvis, femur, tibia, and fibula bone meshes. For each anatomical joint, we used a double animation joint system in which one joint specified the location of the actual joint, and another animation joint (the first joint’s child) could be used to manipulate the rotations at that joint. These animation joints will be referred to as the position animation joint and the rotation animation joint. The orientation and location of these animation joints were the same as the joints set up for the osteological RoM study (see above).

For each joint, we created an alpha shape in MATLAB R_2019b (Mathworks, Natick, MA, USA) from the viable poses from the osteological joint RoM analysis (where FE rotations were graphed on the *X-*axis, ABAD rotations were graphed on the *Y*-axis, and LAR rotations were graphed on the *Z-*axis) (Manafzadeh and Padian 2018). The alpha radius was determined by plotting the points and alpha shape on the same graph and adjusting the alpha radius until the alpha shape wrapped all of the viable poses (without including unviable poses [see the "Results" section]). The alpha mesh was then imported into Maya as an .obj file (code for graphing points, alpha shapes, and exporting as .obj available on Github; see the "Data Availability" section). Note that alpha shape meshes must be clean (no non-manifold or self-intersecting faces, with uniform normals) in order for the method to work properly.

To enable comparisons between the salamander hindlimb poses used during walking and the *E. megacephalus* models, we scaled the *S. salamandra*model such that the distance between the centroid of the acetabular ACS to the distal tibia/fibula in the null pose was consistent between models. This scaling step was done separately for the two knee options. The measurement point on the distal tibia/fibula was determined as the intersection point of the LAR axis and a plane aligned to the distal-most points on the tibia and fibula, with the tilt in the FE axis at about 90 degrees to the LAR axis. We then exported the salamander model in the four key configurations chosen above (mid-swing, toe-on, mid-stance, and toward end of the stance) and imported it into the Maya scene with the *E. megacephalus* model. For each configuration, we aligned the salamander model's acetabular ACS to the *E. megacephalus* acetabular ACS with a point and orient constraint, and then manipulated the *E. megacephalus* hip and knee joints to match the *S. salamandra* bones, to achieve gross morphological similarity of the body and limb configuration relative to the ground. Such similarity was defined as aligning the bones as closely as possible to each other. Due to differences in femoral “torsion” (i.e., relationship of proximal and distal articular surfaces) in *E. megacephalus* and *S. salamandra*, exact alignment of all bony features is not possible; in these cases we gave priority to aligning the distal femoral articular surfaces and the articular surfaces of the tibia and fibula. We set the limits of the hip and knee animation joints from 180 to −180 FE, 90 to −90 ABAD, and 180 to −180 LAR to prevent redundant poses that inadvertently exceeded the sampled poses in the osteological RoM analysis.

To check whether these limb positions were viable in *E. megacephalus*, we automatically checked them against the osteological RoM results (via alpha shapes) using a GUI (graphical user interface) called “multiJointPoseChecker.mel” developed for the purpose of checking the validity of several joints at once (Supplementary Fig. S3). These steps were done for both knee spacing options and are described below.

“multiJointPoseChecker.mel” enables the user to input between one and three animation joints and the meshes for corresponding joint rotation alpha shapes. Then, when the user manipulates the joints, the script tests whether the joint rotations fall within the viable pose space; if they do not, the alpha shape turns red. This enables the user to manipulate several joints to achieve specific limb orientations with instantaneous feedback of whether all of the given joint positions are viable.

## Results

**Fig. 5:**
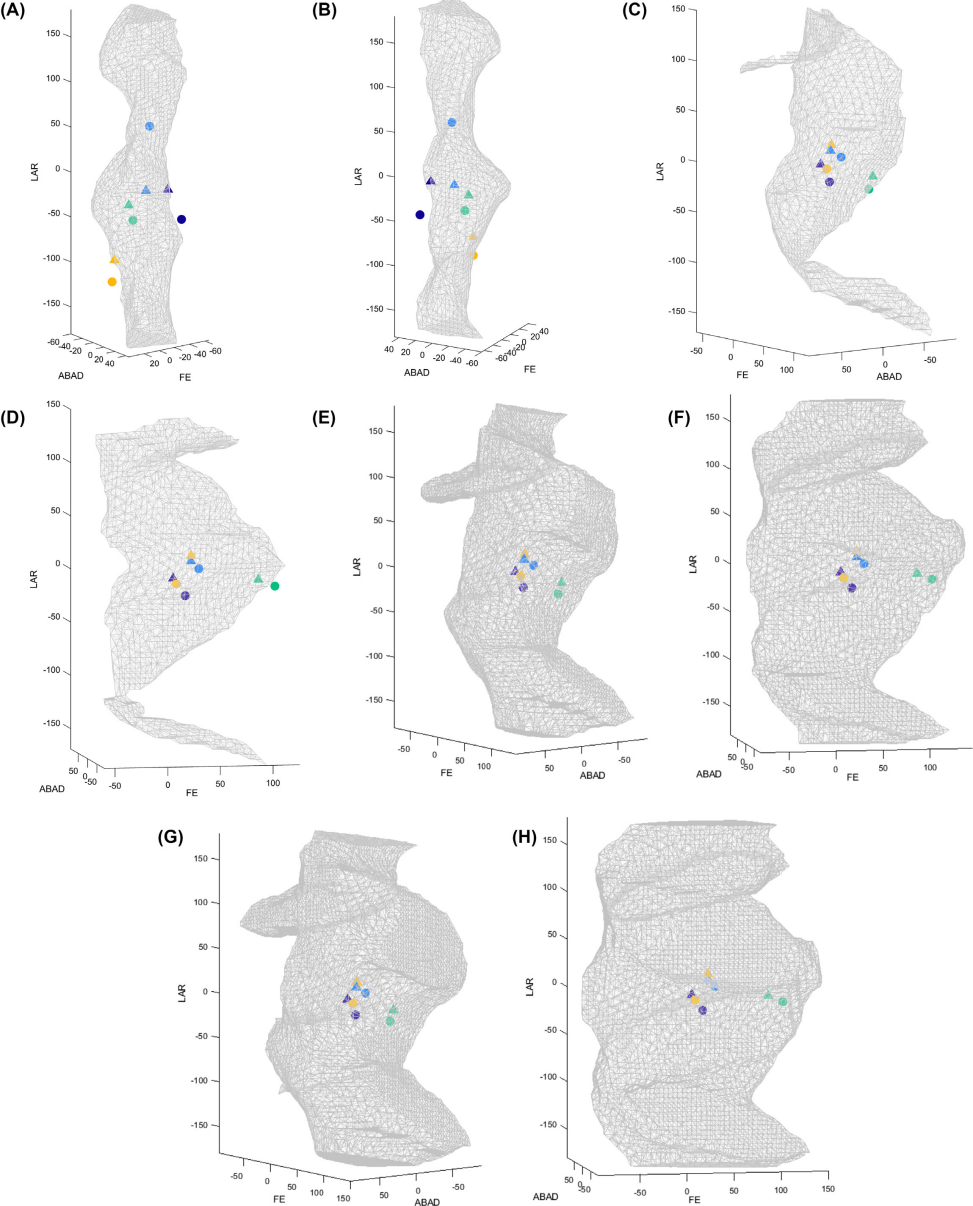
Alpha shapes illustrating osteological RoM for the *E. megacephalus* hip (**A** and **B**) and knee **(C–H)** joints. (C and D) Knee spacing option A (tight spacing); (E and F) knee spacing option B (intermediate spacing); and (G and H) knee spacing option C (large spacing, same relative joint spacing as salamander in null pose). Salamander joint poses used during key phases of the walking stride cycle are depicted as circles, replicated poses in *E. megacephalus* are depicted as triangles. Purple: mid-swing, blue: toe-on, green: mid-stance, and yellow: just before toe-off.

The *E. megacephalus* hip had 8500 unique viable poses out of 186,624 sampled, knee A had 8309 unique viable poses, knee B had 30,628 unique viable poses, and knee C had 48,034 unique viable poses. Euler angle rotations (maximum and minimum rotational values of each axis, and the corresponding rotations about the other axes at these poses) obtained from *E. megacephalus* osteological RoM are shown in Table 2. Alpha shapes are shown in Fig. 5. An alpha shape radius (i.e., “tightness” of wrapping the viable Euler space) of 10 was chosen after iterative tests with various radii to determine the tightest possible bounding value that included all of the points without excluding viable poses. Note that we did not cosine-correct the Euler space (Manafzadeh and Gatesy 2020) for our study (since we were not comparing overall volumes); therefore, direct comparisons of alpha shape volumes and viable percentage of total poses samples do not reflect exact quantitative differences in pose space. However, the differing numbers of viable poses do demonstrate that overall reconstructed osteological joint mobility at the knee was sensitive to the joint spacing (cf. Demuth et al. 2020; Manafzadeh and Gatesy 2021).

**Table 2:** *Eryops megacephalus* osteological RoM.

Joint	Max FE	Min FE	Max ABAD	Min ABAD	Max LAR	Min LAR
Hip	**40**, -20, -40	**-65**, -5, 95	-15, **45**, -155	-30, **-60**, 15	-35, -5, **180**	-35, -5, **-180**
Knee A	**125**, -15, 10	**-60**, -45, 130	15, **90**, 100	0, **-90**, -125	5, -15, **150**	95, -80, **-170**
Knee B	**145**, -45, 30	**-80** -60, 130	-30, **90**, 85	-50, **-90**, -100	-25, -10, **180**	-25, -10, **-180**
Knee C	**150**, -50, 15	**-95**, 0, -25	-55, **90**, 80	-60, **-90**, -100	-45, -5, **180**	-45, -5, **-180**

Knee A is a tight joint space model, knee B is medium joint space, and knee C is a larger joint space (same relative joint spacing as salamander in the null pose). Euler angles for max and min values are shown in bold. The other angles show interaction of degrees of freedom, by showing the rotations about the other two axes at the maxima and minima of the axis of interest. Values are listed in a *Z,Y,X* format, corresponding to FE, then ABAD, then LAR. For example, for maximum FE, the first angle is in bold and the next to values are the ABAD and LAR values to achieve this maximum. For both the knee and hip, positive rotation about the *Z*-axis is flexion, positive rotation about the *Y*-axis is abduction, and positive rotation about the *X*-axis is external rotation. Angle values are relative to a null pose in which the femur extends laterally from the acetabular ACS and the knee is extended, so that the tibia/fibula also extend laterally.

That said, the main goal of this study was not to compare overall mobility but rather to reconstruct salamander-like limb configurations and determine where the joint poses they require fall within the viable pose space. The osteological RoM of *E. megacephalus* permitted four key limb configurations similar to those used in Fire salamanders during walking (Fig. 4). This was true for all three knee spacing options. However, there were some slight differences between the *E. megacephalus* and salamander poses. For mid-swing, the *E. megacephalus* hip was not capable of quite as much femoral protraction relative to the femoral position of the salamander. In this case, we had the option to either position the *E. megacephalus* knee such that the distal tibia and fibula aligned with the *S. salamandra*bones, or recreate the knee joint pose from the salamander in *E. megacephalus*. We opted to reconstruct the latter. Regardless, these slight differences in hip and knee poses likely do not affect the capacity of *E. megacephalus* for salamander-like hindlimb kinematics, because they occur in the non-weight-bearing swing phase.

At the point just before toe-off, the *E. megacephalus* hip could not extend (retract the femur) quite as much as the salamander. For more exact alignment of the femora, the *E. megacephalus* hip would have had to extend about 8 more degrees, which was not possible due to bony intersections at that position. However, with a slight bit of extra knee flexion, the distal ends of the tibia and fibula could be brought into a similar position as in the salamander even with slightly less hip extension in *E. megacephalus*. Furthermore, including translational motions at the joints in the model would likely allow an even more similar pose at the hip at both mid-swing and just before toe-off poses.

Tables 3 and 4 show the hip and knee joint poses used in *S. salamandra* during key configurations used in walking: mid-swing (i.e., maximal anterior excursion of the limb, frame 565), toe-on (frame 636), and mid-stance (frame 716). Tables 3 and 4 also show the hip and knee joint poses in *E. megacephalus* used to replicate the salamander-like limb configurations. Fig. 5 shows the joint poses at the hip and knee associated with these configurations in both *E. megacephalus* and *S. salamandra*, plotted on the alpha shapes.

**Table 3:** Hip joint poses, rounded to the nearest tenth of a degree, listed in the order of FE, ABAD, and LAR for the four key configurations used in this study

	Mid-swing	Toe-on	Mid-stance	Just before toe-off
*S. salamandra*	43.1, 26.2, −46.5	−20.2, −0.2, 54.9	−3.3, −6.9, −48.5	29.0, 0, −109.6
*E. megacephalus* A	−29.2, 20.6, −12.3	−16.3, −1.2, −16.8	0.1, −9.7, −32.0	24.3, −2.3, −86.9
*E. megacephalus*B	−29.2, 20.6, −12.3	−16.3, −1.2, −16.8	0.1, −9.7, −32.0	24.3, −2.3, −86.9
*E. megacephalus* C	−29.2, 20.6, −12.3	−16.3, −1.2, −16.8	0.1, −9.7, −32.0	24.3, −2.3, −86.9

**Table 4:** Knee joint poses, rounded to the nearest tenth of a degree, listed in the order of FE, ABAD, and LAR for the four key configurations used in this study

	Mid-swing	Toe-on	Mid-stance	Just before toe-off
*S. salamandra*	32.1, −3.9, −17.5	44.1, −9.1, 8.2	120.2, 13.7, −11.8	23.1, −7.2, −6.0
*E. megacephalus* A	20.8, −1.6, −1.2	38.0, −0.7, 14.5	101.1, −5.3, −3.5	38.0, −1.9, 20.2
*E. megacephalus* B	20.8, −1.6, −1.2	38.0, −0.7, 14.5	101.1, −5.3, −3.5	38.0, −1.9, 20.2
*E. megacephalus* C	20.8, −1.6, −1.2	38.0, −0.7, 14.5	101.1, −5.3, −3.5	38.0, −1.9, 20.2

## Discussion

The osteology of the pelvis and stylopodial and zeugopodial hind-limb bones permitted *E. megacephalus* to achieve four configurations characteristic of four main points in the stride cycle of a salamander: mid-swing, toe-on, mid-stance, and just before toe-off. Overall, our data lend support to the idea that *E. megacephalus* could have moved with (Fire) salamander-like hindlimb kinematics.

### Joint RoM and limb postures

Investigating joint RoM during locomotion in an extant animal can help us understand the coordination of joint positions that enable its gait. As a result, testing for possible gait(s) in a fossil requires not only comparing the joint RoM values between extant and fossil taxa but checking how the various poses at the limb joints interact to produce, for example, an appropriate foot orientation. Joint angles at the hip and knee joint cannot be simply transferred from any animal to another; foot ground contact, hip height, and limb segment lengths impose constraints on possible poses. If two animals have differing limb segment lengths, applying the joint angle of the first animal to the second will probably produce an unfeasible gait in the latter (Gatesy and Pollard 2011). Similar limb proportions are important when comparing joint angles to infer overall limb and body position relative to the ground (Gatesy and Pollard 2011). However, the morphology of the segments is still important; the same combination of rotations at the hip and knee joint in both animals could result in a different foot orientation on the ground given differences in torsion between the proximal and distal articular surfaces of a bone (Gatesy and Pollard 2011; Carney 2016).

Indeed, there were some salient differences in the morphology of *E. megacephalus* and *S. salamandra*. These differences did not prevent similar overall poses but instead highlight that combinations of different morphologies can produce similar overall limb configurations (and by inference, locomotor patterns) in different animals. For example, the shapes of the proximal femur in *E. megacephalus* and *S. salamandra*differed. In the null pose with the leg laterally extended, the longest dimension of the femoral heads of the two animals were roughly 90 degrees offset from each other. In order words, with the limb extended and the longest dimension of the distal articular surface aligned between the two models, the salamander proximal femur had a more antero-posteriorly compressed shape, in contrast to the more dorsoventrally compressed proximal femur of *E. megacephalus* (Fig. 2). Differences in hip LAR (Table 3 and Fig. 5) between *S. salamandra* and *E. megacephalus* can be attributed to the difference in this femoral “torsion” (the relationship between proximal and distal articular surfaces). This leads to more measured internal rotation in hip LAR in *S. salamandra* relative to *E. megacephalus* for a visually similar proximal femur orientation. However, while *S. salamandra* did have higher hip internal rotation (Table 3 and Fig. 5), it is not as large as this 90 degree morphological offset, because we prioritized matching the distal femur between the two taxa over exactly matching the proximal femur given the distal femur’s importance in setting up the functional axis of the knee joint. The benefit of the new method we present here is that it facilitates pose comparisons despite differences in morphology and ACSs (see the “Methodology” section).

Another difference between the two taxa is the morphology of the distal fibula. The distal fibula in *E. megacephalus* does not reach as distally as the distal tibia in *E. megacephalus*. In the salamander, on the other hand, the distal tibia and fibula were about the same height relative to the ground. However, despite this difference, the two animals could still have had a similar stance phase position of the foot. An articulated fossil of the distal tibia and fibula and tarsals of *E. megacephalus* shows that the intermedium and fibulare were large in *E. megacephalus* and articulated with the distal fibula (Dilkes 2015, Fig. 6). The distal articular surface of the fibulare roughly aligns with the distal surfaces of the tibiale and centrale 4 ([Bibr bib10][Bibr bib10]), forming a hinge-like flexion axis with the distal centralia and fourth and fifth tarsals, and another flexion region between the distal tarsals and metatarsals (as described for the Permian tetrapod *Trematops* in Schaeffer 1941). Therefore, although the distal tibia and fibula were offset, these ankle and foot bones could have permitted a “hinge-like” flexion in the foot to place the plantar surface of the foot on the ground. Future studies could test this by modeling *E. megacephalus* foot and ankle mobility and rotoscoping the salamander foot and ankle joint, to add possible foot positions to the limb configurations tested in this study.

**Fig. 6:**
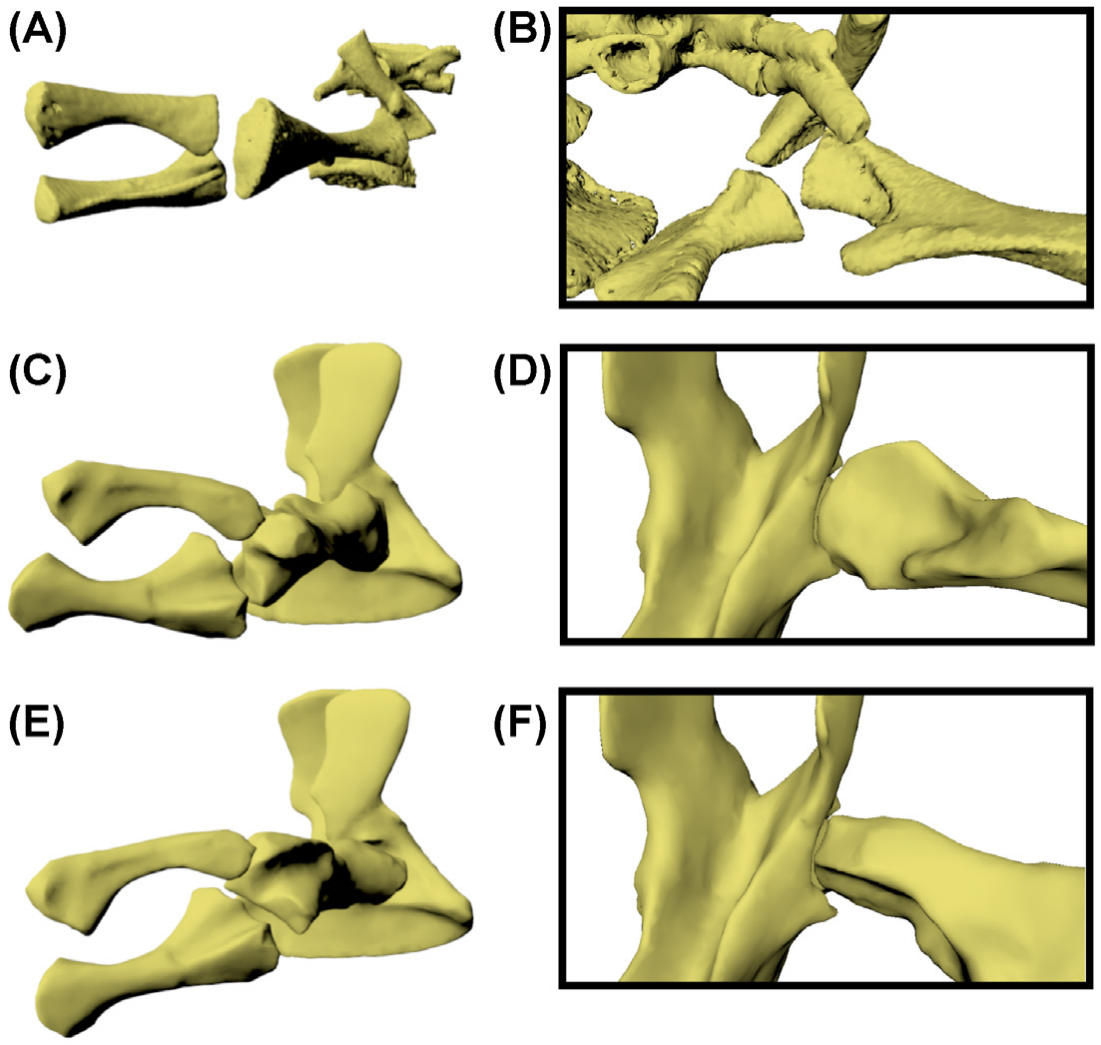
Hindlimb pose just before toe-off for *S. salamandra***(A,B)** and *E. megacephalus***(C-F)** in lateral (A, C, and E) and posterior (B, D, and F) views of the hip joint. C and D show the version of the *E. megacephalus* hindlimb from Fig. 4 (with intermediate knee joint spacing option), with the limb bones aligned as closely as possible to those of *S. salamandra*. E and F show an alternative just before toe-off pose for *E. megacephalus*, with less internal rotation of the hip, resulting in a hip joint where the proximal femoral surface is more aligned with the acetabular shape.

Furthermore, since foot positioning likely involves some movement of the fibula relative to the tibia (Schaeffer 1941), future models could incorporate this additional movement to analyze the interaction of the crus and foot position. Such studies may help elucidate the mechanism in which the foot faced anteriorly during retracting of the femur in sprawling locomotion to produce forward propulsion during femoral retraction (e.g., the “rotation problem” discussed for lizards in Rewcastle 1983). An anteriorly facing foot may be achieved via different mechanisms in lizards than in *E. megacephalus* and *S. salamandra*. The lizard ankle joint differs from that of amphibians and early tetrapods; lizards fuse some of their tarsals (see Schaeffer 1941 for an extensive description of amphibian and reptile tarsal anatomy). In *E. megacephalus* and *S. salamandra*, the morphology of the tarsals (including a relatively large intermedium, fibulare, and centrale) may facilitate the forward foot orientation, but further studies are needed to test this hypothesis.

### Early tetrapod locomotion

Our study contributes to a growing literature examining the locomotor capabilities of early tetrapods. When Pierce et al. (2012) used a posture-based approach to compare the Devonian tetrapod *Ichthyostega* to salamanders, they concluded that *Ichthyostega* could not have achieved a sprawling mid-stance pose similar to that of a Tiger salamander *(Ambystoma tigrinum*), and that the hip had much lower LAR capacity than modern tetrapods. By contrast, our RoM studies show that the Permian tetrapod *E. megacephalus* has unrestricted LAR when the limb is extended laterally; however, with high hip extension (retraction) such as at toe-off, our inferences of LAR also become a bit more restricted. No studies to date have quantitatively compared the appendicular morphology or mobility of stem tetrapods and later tetrapods such as *E. megacephalus* on a like-for-like basis to address just how similar or different the joint form and function were. As a result, future comparative studies using similar null poses and joint mobility methods are needed to investigate how variation in early tetrapod morphology relates to locomotor function, whether certain salamanders are better analogs for locomotor analyses in some early tetrapods than others, and why.

Our inferences of hip LAR are likely sensitive to hip joint spacing. We did not test a model of *E. megacephalus* with less joint spacing at the hip, but such a model could have resulted in more restriction of LAR. Specifically, the supra-acetabular buttress may constrain internal rotation of the femur. Salamanders also have this buttress, but relative to the distal femur, the longest dimensions of the proximal femoral articular surface are offset by about 90 degrees in salamanders and *E. megacephalus*; in the toe-off position, the *E. megacephalus* proximal femur has the longest dimension dorsoventrally, whereas the salamander femur has the longest dimension anteroposteriorly. Therefore, in mid stance and end of stance poses (Figure 4), the buttress does not restrict the femoral head as much in *S. salamandra* (when the distal femoral articular surfaces are aligned between *E. megacephalus* and *S. salamandra*). On the other hand, allowing a range of translations in all three degrees of freedom will likely permit more mobility in *E. megacephalus* (Manafzadeh and Gatesy 2021), requiring future sensitivity analyses.

As discussed above, the *E. megacephalus* limb configuration just before toe-off was possible, but the femoral head in this position has its longest dimension dorsoventrally, rather than the more anteroposteriorly oriented acetabulum, and the space between the femur and supraacetabular buttress is very small (Fig. 4D, I, M, and Q). Therefore, we also tested another possible toe-off configuration in *E. megacephalus* (Fig. 5E and F). In this configuration, we allowed for more variation between *E. megacephalus* and *S. salamandra*, with the *E. megacephalus* femur less internally rotated to better align the femoral head and acetabulum in *E. megacephalus*. Such a pose is less constricted at the hip joint; therefore, it is less sensitive to hip joint spacing. It results in the whole limb being oriented a bit more anteriorly and a bit less internally rotated than in the salamander. However, the relative positions of the distal tibia and fibula relative to the body are not very different from those of the salamander (Fig. 5A, B, E, and F). Therefore, we conclude that even if *E. megacephalus* exhibited less internal rotation than the salamander at the end of stance (Fig. 6E and F), it could have had a similar sprawling movement to a salamander, but probably varied in details of the movement, such as stride length. Further tests of articular and soft tissue constraints might elucidate which toe-off position is more likely, but regardless, both options support our hypothesis that salamander-like hindlimb kinematics were permitted by the osteological RoM of *E. megacephalus*.

### Methodology

One big advantage of the new method we present here is that it avoids confusion resulting from ascribing homology to different anatomical axes in different taxa, since we are not directly comparing Euler angles between two taxa. Instead, we take the series of limb configurations used by an extant animal for a specific gait, and replicate those overall limb configurations in the fossil, then test the joint poses required for each configuration against the boundaries of the osteological RoM in the fossil. Although the potential poses could have been checked visually for bone-on-bone interpenetrations, this new approach enables direct visualization of joint rotations within their respective possible pose spaces and simultaneous objective assessment of their viability, and is especially useful for “tight” joints such as the hip or vertebral elements, where visual checks are difficult because the bones often obscure the joint morphology. Furthermore, the method could be used to enforce joint mobility limits (including interaction of degrees of freedom) in digital and robotic simulations of whole limb and even whole body movement. Although bony collisions could also be measured in such simulations via Boolean intersections between the bones, our method could be used with alpha shapes generated from joint RoM including soft tissues to enforce joint RoM limits beyond bony intersections.

### Future directions

We demonstrated that, based on osteological joint RoM, *E. megacephalus* could have adopted the hindlimb configurations used in the sprawling gait of a Fire salamander, suggesting that certain salamanders may yet be useful analogs for early tetrapod locomotion in some cases.

Our study is one example of where the choice of “analog” (or homolog) is very important to consider carefully, and specifically. For example, it should be explicitly stated what is being tested as potentially analogous—for example, mobility of a particular joint or similarity of overall limb configurations, as in this study, or other aspects of locomotor biomechanics such as muscle function or kinetics. It may be unlikely that all aspects of locomotion are analogous between an extinct taxon and an extant taxon, given the complex mosaic of potential differences between them in overall 3D morphology and dynamics. Salamanders may not be good analogs for certain aspects of locomotion in early tetrapods, especially in highly specialized forms such as *Crassigyrinus* (Clack 2002b; Herbst and Hutchinson 2018); or early amniotes such as *Orobates* (Nyakatura et al. 2019).

Early tetrapods also varied dramatically in adult body sizes, from small sizes closer to that of the Fire salamander in this study to larger sizes ∼2 m in length (e.g., Clack 2002a). Limb posture in vertebrates on land tends to get more erect/upright with increasing body size in order to satisfy biomechanical constraints such as supportive tissue stresses (Dick and Clemente 2017). Hence, the issue of body size and limb kinetics deserves consideration in conjunction with analyses of kinematics.

Ideally, future studies could build on our passive joint RoM studies. This range of poses could be further narrowed down, for example, by following the kinetic (biomechanical) constraints described in Gatesy et al. (2009) and Nyakatura et al. (2019) or the ligament modeling approach described in Manafzadeh and Padian (2018). Our study only focused on the hindlimb—future models could also include the forelimb as well as spine kinematics, which play an important role in salamander locomotion (Ashley-Ross 1994).

Musculoskeletal models of *E. megacephalus* and other early tetrapods could be made based on muscle reconstructions (e.g., those discussed in Molnar et al. 2018, 2021), to thoroughly test the scope of possible motions and whether the large size of *E. megacephalus* would impose kinetic (as opposed to kinematic) limits on salamander-like motions. Notably, the trochanter on the salamander femur and the trochanters on the *E. megacephalus* femur (fourth and internal trochanter [Pawley and Warren 2006]) were in similar positions throughout the stride cycle. This suggested similarities in muscle attachment and function, and more detailed examination of osteological correlates and their location throughout the stride could enable the modeling of muscles.

Furthermore, there is the potential to further develop our new multi-joint pose viability methods. For example, this workflow would benefit from automatic methods to best match up bone meshes, or align anatomical landmarks automatically (e.g., via algorithms similar to the software CloudCompare; https://www.cloudcompare.org/), to objectively ensure that specific osteological correlates—and therefore the muscles that drive the limb motion—are aligned between animals. Additionally, all three translational degrees of freedom could be included in the model and incorporated into the pose viability analysis to test their effects on potential limb configurations Manafzadeh and Gatesy (2021). Further studies in extant animals on joint spacing (e.g., Molnar 2021; Holliday et al. 2010) will help to inform the relationships between bone and cartilage morphology and size in extant species and to determine how much joint translation to allow.

Moving forward, additional analyses of early tetrapod locomotion will benefit from the development of more objective articulation criteria in evaluating pose viability (Manafzadeh and Gatesy 2022). For example, in this study, full extension of the knee was possible in the *E. megacephalus* model, but the tibia and fibula were substantially more dorsal than the femur (i.e., potentially subjectively “disarticulated”). Determining when to assess such poses as viable or inviable will strongly influence the inferences we draw about which gaits were possible. The work presented here offers a foundation for these future analyses.

## Conclusion

Here, we provide insight into the possible locomotor mode of *E. megacephalus*, demonstrating that its osteological joint anatomy did not prohibit a salamander-like sprawling hindlimb gait. Our new multi-joint pose viability method provides a new tool to test whether a fossil is capable of achieving the overall limb postures characteristic of a certain gait. It can readily be applied to other taxa to examine the limb postures that could have been achieved across a range of taxa. Together with soft tissue reconstructions, this will enable us to better understand the major locomotor transitions in vertebrate evolutionary history.

## Funding

This work was funded by the Natural Environment Research Council NE/K004751/1 to JRH.

## Supplementary Material

icac083_Supplemental_File

## Data Availability

MEL script for the multiJointPoseChecker GUI is available via the XROMM Mel scripts Bitbucket (*https://bitbucket.org/xromm*) in the XROMM_other_MEL_scripts repository. Matlab script for creating alpha shapes, plotting poses on alpha shapes, and exporting alpha shapes as.objs is available on Github; the latest Github release can be accessed via *https://doi.org/10.5281/zenodo.6573956*.

## Supplementary data

Models of *E. megacephalus* at original specimen size are available on Morphosource and the Field Museum EMu data repository (pelvis: *https://doi.org/10.17602/M2/M443518, https://db.fieldmuseum.org/f771c09a-886d-41e9-87f6-c556b40ca9cb*; femur: *https://doi.org/10.17602/M2/M443523, https://db.fieldmuseum.org/eb93cc57-6dff-4844-b528-c53a7732764e*; fibula: *https://doi.org/10.17602/M2/M443528, https://db.fieldmuseum.org/9013a426-c67e-47bf-be13-77188714800e*; tibia: *https://doi.org/10.17602/M2/M443533, https://db.fieldmuseum.org/ad2923fa-7f29-4fa3-b6fd-a02fcd9ef8c0*).

If using code or models from this paper, please cite this paper as well as referencing the relevant code repository or model DOI.
