# Supplementary material for: Multi-Joint Analysis of Pose Viability Supports the Possibility of Salamander-Like Hindlimb Configurations in the Permian Tetrapod Eryops megacephalus
Source: Integr Comp Biol. 2022 Jun 10;62(2):139–51. doi: 10.1093/icb/icac083 (PMC9405718; doi:10.1093/icb/icac083)
Supplement: icac083_Supplemental_File [file icac083_supplemental_file.pdf]

Supplementary Info

Supplementary Text 1: *Eryops megacephalus* model preparation

In Meshlab, we downsampled each mesh and scaled the mesh, using a 10 cm scale bar that we included in the photogrammetry model background for this purpose. We measured the dimensions of this scale bar (= x) and then scaled the mesh by a factor of 10/x, so that the model dimensions of the scale bar were 10 cm. This ensured that the dimensions of the model were equal to the real dimensions of the bone. After both halves of each object were cleaned, downsampled and scaled, we aligned the two halves of each element using CloudCompare software (by picking shared points in both meshes) (CloudCompare version 2.5.4.1, GPL software, retrieved from http://www.cloudcompare.org/). We exported the aligned meshes, merged the two halves, and resampled to create a watertight mesh in MeshLab software (poisson surface reconstruction, Meshlab v1.3.3, Cignoni et al. 2008). Then, we scaled the models based on the ratios obtained from the literature (Table 1), keeping the pelvis at the actual size and scaling the other bones to that individual (Table S2). Measurements and scaling were performed in MeshLab software. The absolute bone sizes do not influence joint range of motion studies; only the relative sizes of the elements must be correct. Details of the scaling are given below. The final scaled models of the *E. megacephalus* hindlimb are displayed in Figure 1.

We chose the most complete, well-ossified pelvis, FMNH UC 446 (Fig 1 A,B). We chose femur FMNH UC 33 because it had the best preserved articular surfaces. On the proximal femur, the anterior caput femora fossa and posterior caput femora fossa (described in Pawley and Warren 2006) are present in this specimen but not the other femora in the collections (Fig. 1 C,G). Only one fibula was complete in the FMNH and UMZC collections, specimen FMNH UC 203 (Fig. 1 D,H). Two tibia specimens were well preserved, so we chose the larger tibia, FMNH UC 1250 (Fig 1 E,F), because its size most closely matched the size of the fibula (i.e. these bones probably came from similarly sized individuals). To ensure that the final model represented a realistic individual (not a chimera of juvenile and adult elements; imposing potential allometric or other ontogenetic shape/size biases), we chose specimens that did not appear to be juveniles, based on size and ossification.

We used Pawley and Warren 2006 as a reference guide for the relative sizes of the femur and tibia. The descriptions and illustrations in this paper are based on well-ossified (i.e. presumably mature) specimens at the American Museum of Natural History, New York (AMNH) and Museum of Comparative Zoology, Cambridge (MCZ). We measured the AMNH 4203 femur and tibia in the specimen photos (citealtPawley2006a figure 10 and 11). The shared specimen number of this femur and tibia indicates that these bones likely belong to a single individual, so we used this femur size to tibia size ratio to scale the bones. We used this ratio to linearly scale our photogrammetry models from the FMNH collections (Table 1).

The pelvis photographed in the Pawley and Warren paper is not the same individual as the tibia and femur. To determine the most plausible proportions of the femur to pelvis size, we used plate IV in Cope 1880 for reference. This illustration depicts a femur and pelvis from the same individual, at the same scale, and the ratio of the length of the femur to the craniocaudal length of pelvis was 0.9 (the dimensions selected for measurement are arbitrary, as long as we used them consistently for our models, so we chose the dimensions easiest to measure). For the tibia to fibula ratio, we used the maximal proximodistal lengths of these bones, reported in Cope 1888. This information from the literature gave us the limb bone proportions for an individual (Table 1).

Scaling of models

The femur needed to be scaled the most (1.24x) to fit the pelvis; in other words, the original femur bone came from a smaller specimen than the other elements. However, the articular surfaces were well-ossified and the morphology corresponds to the femoral anatomy described in Pawley and Warren (2006). The tibia and fibula only needed a slight scale adjustment (Table S1), indicating that the pelvis, tibia, and fibula stem from similarly sized individuals.

To assign an anatomical coordinate system to the right acetabulum, we used geometric primitive fitting methods modified from those described in Herbst et al. (in review). The *E. megacephalus* FMNH UC 446 pelvis was distorted due to taphonomic effects, and the left and right acetabula were not symmetrical about the midline. Therefore, using the method of fitting a line through the centroids of ellipsoids fitted to the left and right acetabulum would have created a mediolateral axis that was skewed (yawed) relative to the sagittal plane. To resolve the deformation in the specimen, we used the right side as reference, because it is less mediolaterally compressed than the left side, and the mediolateral dimensions of the right side appear similar to those to other specimens depicted in the literature [Cope, 1880, Pawley and Warren, 2006].

We segmented out the right acetabulum: the convex articular surface bordered posteriorly by the postacetabular and supracetabular buttress (buttresses described in Pawley and Warren 2006. We fit both an ellipsoid and a sphere to this articular surface using Matlab code from Bishop et al. [2021]. The ellipsoid fit was selected for use because it had a similar curvature to and more overlap with the segmented articular surface of the acetabulum than the sphere fit did. The centroid of the ellipse formed the origin of the right acetabular ACS. We then created a sagittal plane, bisecting the pelvis into left and right halves, by fitting a plane through three points on the pelvic symphysis (anterior-most, posterior-most, and ventral-most) in Rhino software (Fig. S1 B,C). We then used this plane to mirror the right pelvic ellipsoid to the left side, to account for the compression on the left side and reconstruct where the left acetabulum would be without the deformation (Fig. S1 D).

We drew a line between the left and right ellipsoid centroids to create a mediolateral axis that was symmetrical about the midline (Fig. S1 D). The antero-posterior axis was drawn between the anterior and posterior points on the pelvic symphysis, and we translated this axis to the midpoint of the mediolateral axis (Fig. S1 E). Because these points were the same ones used to create the midline plane and the acetabular centroid was mirrored across this plane, the mediolateral and anteroposterior axes were already perpendicular (Fig. S1 E).

We then calculated the dorsoventral axis as normal to the plane formed by the mediolateral and anteroposterior axes (i.e. orthogonal to the other axes) (Fig. S1 E). Finally, we translated these anatomical axes to the centroid of the right acetabular ellipse to create the right acetabular ACS (Fig. S1 F). Axis directions for the acetabular ACS corresponded to the hip axis directions of the salamander pelvis in Herbst et al. (in review). The dorsoventral axis of the acetabular ACS was the Z (FE)

axis and pointed ventrally (Fig. S1 F). The anteroposterior axis of the acetabular ACS was the Y (ABAD) axis and pointed posteriorly. The mediolateral axis of the acetabular ACS was the X (LAR) axis and pointed laterally.

For the proximal and distal femur ACSs, we used the same point-based methods in Rhino as described in Herbst et al. (in review). For the salamander studies in Herbst et al. (in review), we kept the two elements connected and scanned them with microCT in that articulation, so we knew their relative positions and spacing in life. However, for *E. megacephalus* the tibia and fibula were isolated elements. Therefore, we needed to set up their relative poses before we could place the points in Rhino and calculate the proximal tibia/fibula ACS. To do this, we imported the tibia, fibula, and femur into Maya. Using the tibial and fibular articular surfaces (described in Pawley and Warren 2006) on the distal femur as a reference for the spacing between the tibia and fibula. We aligned them to the femur by estimating a plausible, anatomically achievable pose (the articular surfaces of the femur face ventrally, so this pose was flexed relative to the (more implausible) null pose of a fully extended limb). From now on, this plausible pose will be referred to as the "resting pose" (*sensu* Otero et al. 2017). We also checked our model spacing against figure 6 in Dilkes (2015), which shows the distal tibia and fibula articulated with the tarsals of *E. megacephalus*. We then exported the tibia and fibula in their new positions (aligned to each other) to create an ACS for the proximal tibia/fibula in Rhino, using the point fitting method described in Herbst et al. (in review).

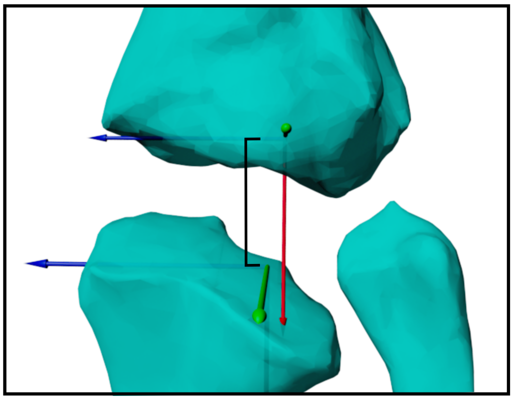

Fig. S2. Determining the joint space in *E. megacephalus* right knee joint, by measuring the distance in the X axis along the distal femoral ACS from the surface of the distal femur to the surface of the proximal tibia.

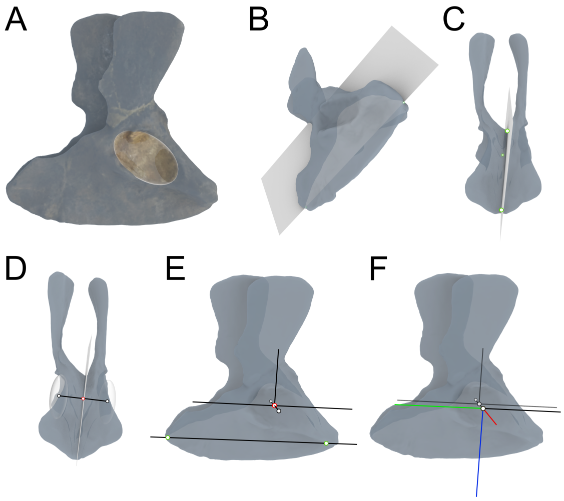

Fig. S1. Creating the right acetabular ACS for the *E. megacephalus* hip. A) Right acetabulum of FMNH UC 446, lateral view, with ellipsoid fit to the acetabulum; B,C) sagittal plane through midline of the body, based on 3 points placed on the pubic symphysis (anterior, posterior, and ventral-most points); D) the right acetabular ellipse was mirrored to left side across midline plane, mediolateral axis formed between centroids of left and right ellipses; E) the anteroposterior axis was drawn between anterior and posterior points on pelvic symphysis, translated to midline of mediolateral axis, and the dorsoventral axis was drawn as the axis orthogonal to the mediolateral and anteroposterior axes; F) pelvis anteroposterior, mediolateral, and dorsoventral axes translated to origin of right acetabulum centroid, to create the right acetabular ACS, with the Z axis (FE, blue) pointing ventrally, the Y axis (ABAD, green) pointing posteriorly, and the X axis (LAR, red) pointing laterally.

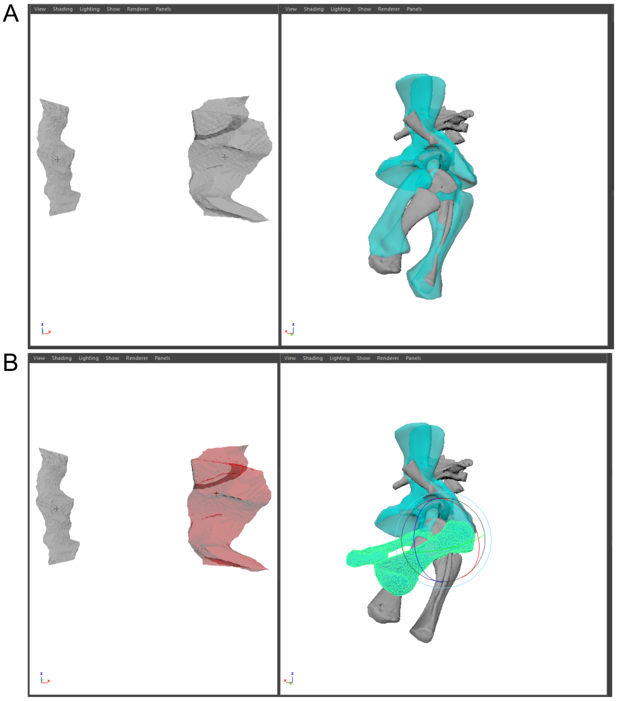

Fig. S3. Method for testing limb orientations and associated joint pose viability. *E. megacephalus* limb in turquoise, *S. salamandra* limb in grey. Alpha shapes on the left characterise osteological range of motion at the hip and knee joints. A) Mid-swing pose recreated in *E. megacephalus*. The alpha shapes on the left remain grey, which indicates that the limb orientation is possible. B) Example of an impossible knee pose; the joint intersections are indicated by the knee alpha shape turning red. The alpha shapes are plotted with the FE axis in Maya global X, ABAD in Maya global Y, and LAR in Maya global Z.

Table S1. Scaling *Eryops* hindlimb models (length is maximum length in given dimension)

Element	Specimen Number	Measurement Dimension	Actual Size	Scaling Factor	Model Size
pelvis	FMNH UC 446	anteroposterior length	239.5 mm	1	239.5 mm
femur	FMNH UC 33	proximodistal length	177.6 mm	1.24	221.1 mm
tibia	FMNH UC 1250	proximodistal length	130.35 mm	1.06	137.6 mm
fibula	FMNH UC 203	distal transverse diameter	47.33 mm	1.03	48.5 mm

References

P. J. Bishop, A. R. Cuff, and J. R. Hutchinson. How to build a dinosaur: musculoskeletal modeling and simulation of locomotor biomechanics in extinct animals. *Paleobiology*, 47(1):1–38, 2021. ISSN 00948373. doi: 10.1017/pab.2020.46.

P. Cignoni, M. Callieri, M. Corsini, M. Dellepiane, F. Ganovelli, and G. Ranzuglia. MeshLab: an Open-Source Mesh Processing Tool. In *Eurographics Italian Chapter Conference*, pages 129–136. The Eurographics Association, 2008.

E. D. Cope. Second Contribution to the History of the Vertebrata of the Permian Formation of Texas. *Proceedings of the American Philosophical Society*, 19(107):38–58, 1880.

E. D. Cope. On the Shoulder-Girdle and Extremities of Eryops. *Transactions of the American Philosophical Society*, 16(2): 362–367, 1888.

D. Dilkes. Carpus and tarsus of Temnospondyli. *Vertebrate Anatomy Morphology Palaeontology*, 1(1):51, 2015. doi: 10.18435/b5mw2q.

A. Otero, V. Allen, D. Pol, and J. R. Hutchinson. Forelimb muscle and joint actions in Archosauria: Insights from Crocodylus johnstoni (Pseudosuchia) and Mussaurus patagonicus (Sauropodomorpha). *PeerJ*, 2017(11), 2017. ISSN 21678359. doi: 10.7717/peerj.3976.

K. Pawley and A. Warren. The appendicular skeleton of Eryops megacephalus Cope, 1877 (Temnospondyli: Eryopoidea) From the Lower Permian of North America. *Journal of Paleontology*, 80(3):561–580, 2006. ISSN 0022-3360. doi: 10.1666/0022-3360(2006)80[561:TASOEM]2.0.CO;2.
